# Over-Expression of Leptin Receptors in Hypothalamic POMC Neurons Increases Susceptibility to Diet-Induced Obesity

**DOI:** 10.1371/journal.pone.0030485

**Published:** 2012-01-20

**Authors:** Kevin M. Gamber, Lihong Huo, Sangdeuk Ha, Joyce E. Hairston, Sarah Greeley, Christian Bjørbæk

**Affiliations:** Division of Endocrinology, Diabetes, and Metabolism, Beth Israel Deaconess Medical Center and Harvard Medical School, Boston, Massachusetts, United States of America; University of Ulster, United Kingdom

## Abstract

Diet-induced obesity (DIO) in rodents is characterized by impaired activation of signal-transducer and activator of transcription 3 (STAT3) by leptin receptors (LepRb) within the hypothalamic arcuate nucleus. This signaling defect likely plays an important role in development of DIO. However, the neuro-chemical identity of the leptin-STAT3 resistant arcuate neurons has not been determined and the underlying mechanisms responsible for development of cellular leptin resistance remain unclear. To investigate this, we first measured arcuate gene expression of known key signaling components of the LepRb signaling pathway and tested whether specifically the critical arcuate pro-opiomelanocortin (POMC) neurons are resistant to LepRb-STAT3 signaling in mice given a high-fat-diet (HFD) compared to mice provided a low-fat control diet (LFD). We found that leptin-dependent STAT3 phosphorylation was decreased within POMC neurons of HFD mice. In addition, *Leprb* mRNA and suppressor of cytokine signaling 3 (*Socs3*) mRNA were elevated in the arcuate of HFD mice. To investigate whether increased LepRb expression *per se* in POMC neurons can influence development of DIO and *Socs3* expression, we created mice that over-express LepRb selectively in POMC neurons (POMC-LepRb). No differences in body weight, fat mass or food intake were found between LFD POMC-LepRb mice and LFD controls. Surprisingly, body weight, fat mass and caloric intake of HFD POMC-LepRb mice was markedly higher than HFD control mice. In addition, arcuate *Socs3* mRNA was increased in HFD POMC-LepRb mice compared to HFD controls. These data show that specifically POMC neurons of DIO mice are resistant to STAT3 activation by leptin, indicating that those cells might play a role in development of DIO. Furthermore, over-expression of LepRb selectively in POMC neurons increases susceptibility to the development of DIO. We propose a model where over-reactivity of the leptin-LepRb signaling system in arcuate neurons may play causal a role in development of diet-induced obesity.

## Introduction

Diet-induced obesity (DIO) in rodents is a principal model of human obesity and results from over-consumption of a diet rich in fat (high-fat diet (HFD)). DIO mice display increased caloric intake, body weight and adiposity compared to mice maintained on a low-fat diet (LFD). Leptin is a hormone produced by adipose tissue and normally acts in the central nervous system to inhibit food intake, and reduce fat mass and body weight [1]. However, despite high circulating leptin levels, DIO animals and obese humans are hyperphagic and have increased adiposity. In addition, the anorexigenic and body-weight reducing effects of exogenous leptin are blunted. This is generally termed leptin resistant obesity [2]. The mechanism(s) whereby a HFD causes leptin resistance and obesity however remain unclear. Elucidation of these issues is important for our understanding of central processes that leads to obesity.

Leptin normally acts on neurons in the hypothalamus and in extra-hypothalamic brain-regions [3], [4]. In particular, neurons within the arcuate nucleus of the hypothalamus (ARC) play a key role in leptin's metabolic actions [2], [5], [6], [7], [8]. Significant attention and importance has been given to pro-opiomelanocortin (POMC) neurons that express functional LepRb [3], [9]. POMC neurons produce several neuropeptides, including the anorexigenic α-melanocyte-stimulating hormone (α-MSH) [10]. α-MSH is a ligand for melanocortin–receptors (MC-Rs) and is a potent inhibitor of food intake [11]. A second population of leptin-responsive neurons also located in the ARC co-expresses agouti-related peptide (AgRP) and neuropeptide Y (NPY) [12]. AgRP stimulates appetite by acting as an antagonist of α-MSH at MC-Rs [13]. Together, the POMC-, AgRP- and MC3/4R-expressing neurons comprise the central melanocortin system [10]. Deletion of LepRb specifically from POMC and AgRP neurons in mice lead to mild obesity [14], [15]. Conversely, Cre-mediated re-expression of LepRb selectively in POMC neurons of the *Lepr^db/db^* mice reduces caloric intake and body weight [16]. Direct leptin action via POMC and AgRP neurons is therefore required for normal body weight homeostasis, although it is also clear that additional nuclei/neurons targets are needed to mediate the full complement of leptin actions.

The leptin receptor (LepR) belongs to the cytokine receptor superfamily [2] and signals via a number of downstream pathways, including the Janus kinase 2 (JAK2) and signal-transducer and activator of transcription 3 (STAT3) pathway [17], [18]. Phosphorylated STAT3 (P-STAT3) regulates gene expression, including stimulation of the *pomc* gene in POMC neurons [3]. Suppressor of cytokine signaling 3 (SOCS3) is a critical negative-feedback regulator of LepRb signaling and its expression is increased transcriptionally by P-STAT3 binding to the *Socs3* promoter in LepRb positive neurons, including POMC cells [14], [19], [20], [21], [22], [23]. In addition, protein tyrosine phosphatase 1B (PTP1B or PTPN1) and the related T-cell-related protein tyrosine phosphatase, TC-PTP/PTPN2, are direct cellular inhibitors of JAK2 and STAT3, respectively [24], [25], [26], [27].

DIO rodents are characterized by hyper-leptinemia in conjunction with elevated arcuate *Socs3* gene expression and impaired leptin-dependent activation of P-STAT3 within ARC neurons [28], [29], [30], [31], [32], [33], [34]. This, together with metabolic data from mice with selective over-expression or deletion of SOCS3 from hypothalamic neurons [35], [36] has promoted the idea that SOCS3 is a key mediator of neuronal leptin resistance, although the mechanism whereby a HFD increases SOCS3 expression is yet unclear. Studies also support possible roles for PTP1B and TC-PTP in causing neuronal leptin resistance in the hypothalamus of DIO mice, since altered levels/activity of those proteins, like SOCS3, are also expected to affect leptin-induced STAT3 phosphorylation [24], [25], [37], [38], [39], [40]. Similar to SOCS3, the mechanism whereby the HFD may up-regulate hypothalamic PTP1B and TC-PTP expression remains undetermined, but might involve inflammatory processes or hyperleptinemia [41], [42], [43]. Additional unresolved critical questions include the neuro-chemical identity of the P-STAT3-resistant hypothalamic neurons of DIO rodents, since those cells likely play principal roles in development and/or maintenance of obesity.

Some studies have reported increased *Lepr* mRNA levels in the hypothalamus of DIO rodents [29], [44], [45]. This increase may reflect a mechanism that serves to counteract leptin resistance. Alternatively and more interestingly, increased leptin receptor expression might directly play a causal role in the development of leptin resistance. Since LepRb signaling increases *Socs3* expression, it is at least plausible that hyperleptinemia might increase SOCS3 protein amounts in key target neurons eventually resulting in long-term neuronal leptin resistance. Indeed, we have earlier reported that acute leptin signaling in cell lines induces long-term SOCS3 expression and a parallel prolonged impairment of STAT3 activation by leptin [46]. In further support of such a model of over-reactivity of the leptin-LepRb system in causing obesity, it has been reported that leptin itself (hyperleptinemia) is required for the development of neuronal leptin resistance (impaired STAT3 phosphorylation in response to exogenous leptin) in the ARC of HFD mice [47]. Moreover, mice that chronically over-express leptin paradoxically accumulates fat mass with age and exhibit increased susceptibility to HFD-induced obesity [48], [49]. The effect of hypothalamic over-expression of LepRb on the development of DIO has not been investigated.

We here demonstrate that specifically arcuate POMC neurons of DIO mice are resistant to LepRb-STAT3 signaling, suggesting an important role of those particular neurons in the development and/or maintenance of diet-induced-obesity. Consistent with some previous reports, we also show increased *Socs3* mRNA and *Leprb* mRNA in the arcuate nucleus of DIO mice. Secondly, we found that genetically-driven over-expression of LepRb selectively in arcuate POMC neurons is sufficient to accelerate development of obesity in HFD mice. Based on these data, we propose a model where over-reactivity of the leptin-LepRb signaling system in hypothalamic neurons, including POMC neurons, may play a role in causing leptin resistance and obesity in high-fat fed mice.

## Results

### Diet-induced obesity and cellular leptin resistance in C57Bl/6J mice

**Figure 1:**
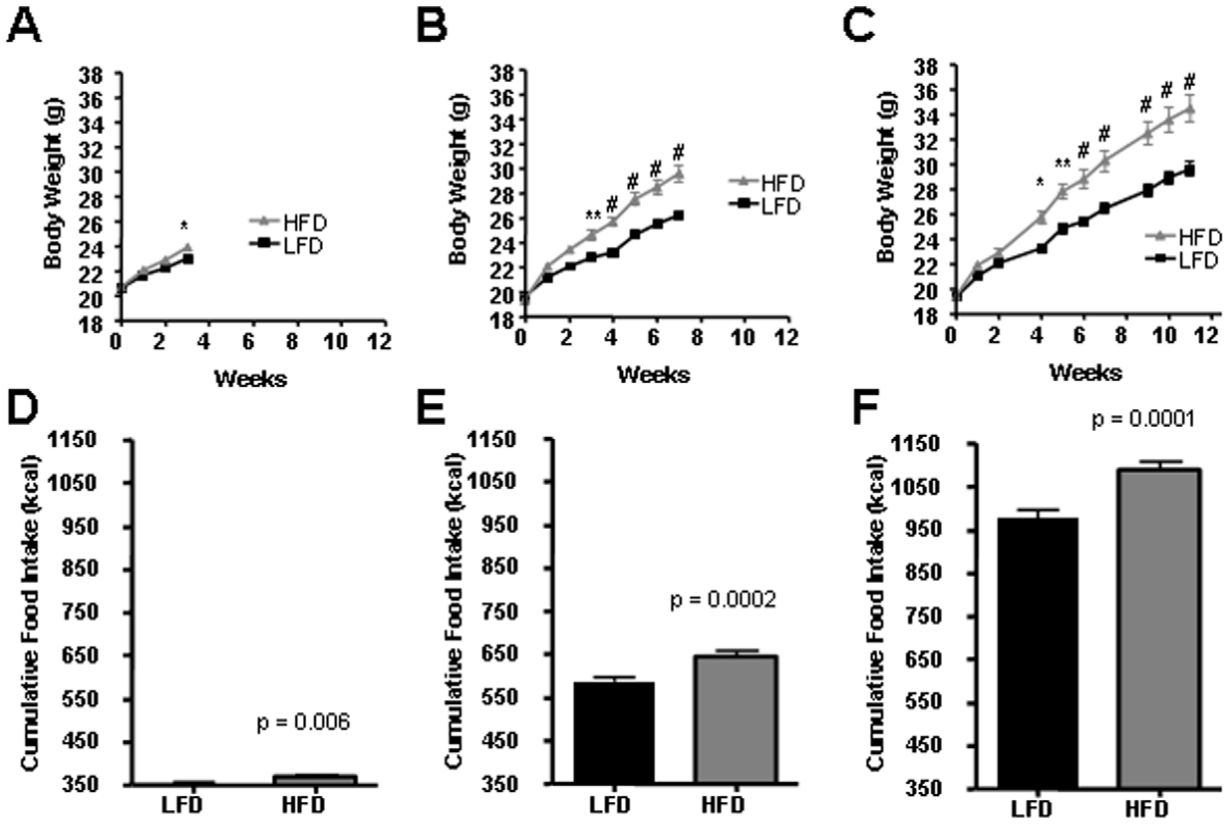
Diet-induced obesity in C57Bl/6J mice. A–C. Shown are body-weight curves of C57Bl/6J mice given HF or LF diets for 3, 7 and 11 weeks, respectively. D–F. Shown is cumulative food intake after 3, 7 and 11 weeks of diets, respectively. * = p<0.05; ** = p<0.01; # = p<0.001. N = 23–25 animals per group. Data are means +/− SEM.

To investigate cell types, genes and mechanisms involved in the development of ARC leptin-resistance in diet-induced obesity (DIO), groups of obesity-prone C57Bl/6J mice were given *ad libitum* access to a high-fat diet (HFD) or low fat diet (LFD) for 3, 7 and 11 weeks. As expected, after 3 weeks, the HFD group had increased body weight (BW) and cumulative caloric intake compared to the animals maintained of a LFD (Fig. 1). Serum leptin levels were not measured, but hyperleptinemia in DIO mice is well documented in previous studies [22], [30], [31], [50]. Differences in BW and food intake between HFD and LFD groups increased further with longer periods of time on the respective diets.

**Figure 2:**
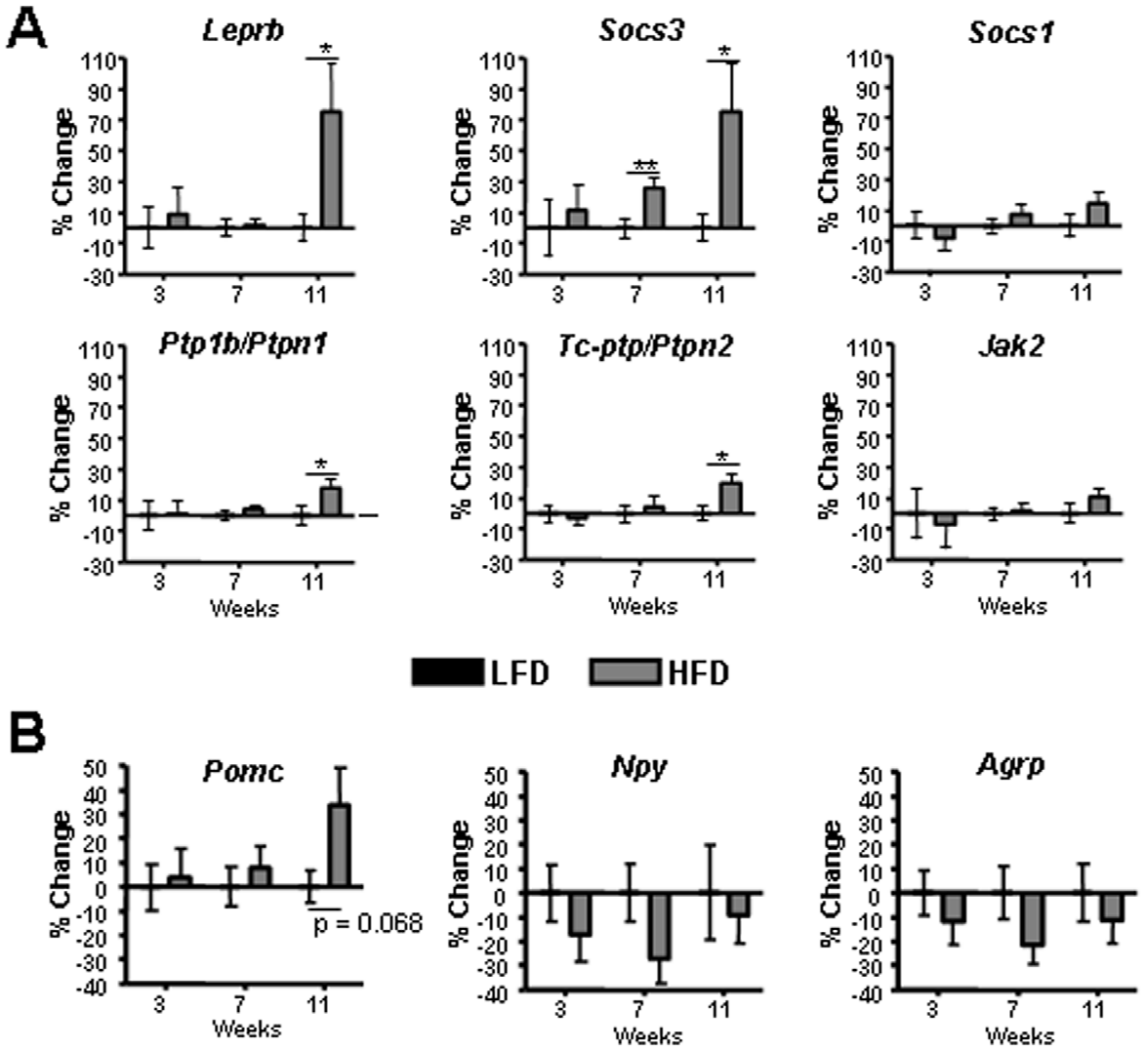
Arcuate nucleus gene-expression in LFD and HFD C57Bl/6J mice. Shown are real-time RT-PCR results from arcuate tissues isolated from LFD and HFD C57Bl/6J mice (Fig. 1). Data for HFD are represented as % change from the LFD control group from each time-point (i.e. 3, 7 or 11 weeks of diets). The average expression level in LFD is normalized to 0% at each time-point. * = p<0.05; ** = p<0.01. N = 22–24 mice per group. Data are means +/− SEM.

To identify genes that might be involved in development of DIO and ARC leptin resistance, mRNA was isolated from ARC tissue of 3-, 7- and 11- week HFD and LFD mice, and subjected to real-time RT-PCR. While most of these genes have been measured earlier, the results differ between studies indicating that further investigations are needed. In addition, few studies have measured the expression at different stages during the development of DIO. We found that *Leprb* mRNA was increased (∼75%) in 11-week DIO mice while *Socs3* mRNA was increased (∼30%) at 7 weeks and further increased (∼75%) after 11 weeks on the HFD (Fig. 2A). Small increases (∼20%) in mRNA levels of the JAK2 phosphatase, PTP1B/PTPN1, and of the PTP1B-related STAT3 phosphatase, TC-PTP/PTPN2, were found after 11 weeks of HFD. Steady-state mRNA levels of *Socs1 and Jak2* were also not different between LFD and HFD groups. Similarly, *Npy and Agrp* levels were not different between groups, although *Pomc* mRNA indicated an increase by ∼35% in 11-week DIO mice, although not reaching statistical significance (Fig. 2B).

**Figure 3:**
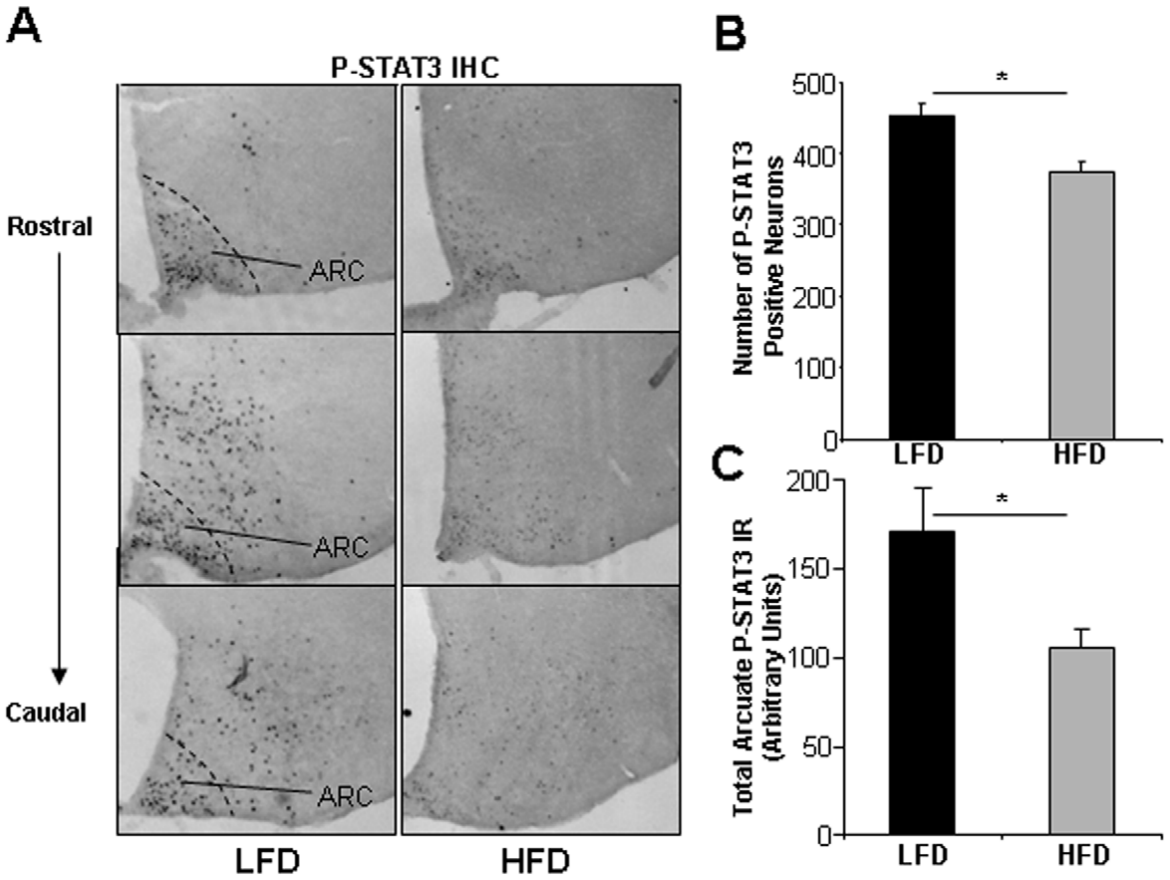
Arcuate neuronal leptin-sensitivity in LFD and HFD C57Bl/6J mice. C57Bl/6J mice were given diets for 10 weeks and injected with leptin (0.6 mg/kg, 30 minutes). A. Shown are representative microphoto images of P-STAT3 immunohistochemistry in the ARC of leptin-treated LFD and HFD mice. Matched representative coronal sections from the rostral, medial and caudal mediobasal hypothalamus are presented. B. Number of P-STAT3-immunoreactive (IR) ARC neurons in LFD and HFD mice. N = 5 sections per animal, and N = 3 mice per group. Counts are from one hemisphere in each section. C. Shown is quantification of the combined immunoreactivity (P-STAT3 IR) of all ARC P-STAT3 positive neurons. N = 5 sections per animal, and N = 3 mice per group. Analyses are done on one hemisphere from each section. * = p<0.05. Arc = hypothalamic ARC nucleus. Data are means +/− SEM.

We have previously reported that the number of P-STAT3-immunoreactive (IR) neurons is reduced in the ARC of leptin-treated DIO C57Bl/6J mice (4- and 16-weeks of HFD) [31], demonstrating cellular/neuronal leptin resistance. To extend those analyses, we here both counted and quantified P-STAT3-IR in ARC neurons in new groups of C57Bl/6J mice given HFD or LFD for 10 weeks. In contrast to previous studies, leptin was only injected at a half-maximal dose (for activation of STAT3)(0.6 mg/kg, i.p., 30 min) according to El-Haschimi et al. [26] to enhance the ability to detect potential differences. As shown in Fig. 3A, P-STAT3 immunoreactivity was indeed reduced in the ARC of DIO mice and we found that the total number of P-STAT3-IR cells was reduced (∼20%) compared to LFD controls (p = 0.013) (Fig. 3B), consistent with earlier data [27], [30]. Furthermore, we show that quantification of P-STAT3 immunoreactivity in all ARC neurons combined was reduced by ∼40% (p = 0.036) in DIO mice (Fig. 3C). Thus, both the total number of leptin-responsive ARC neurons and the signaling responsiveness within individual P-STAT3 positive cells are markedly reduced in leptin-treated DIO mice.

**Figure 4:**
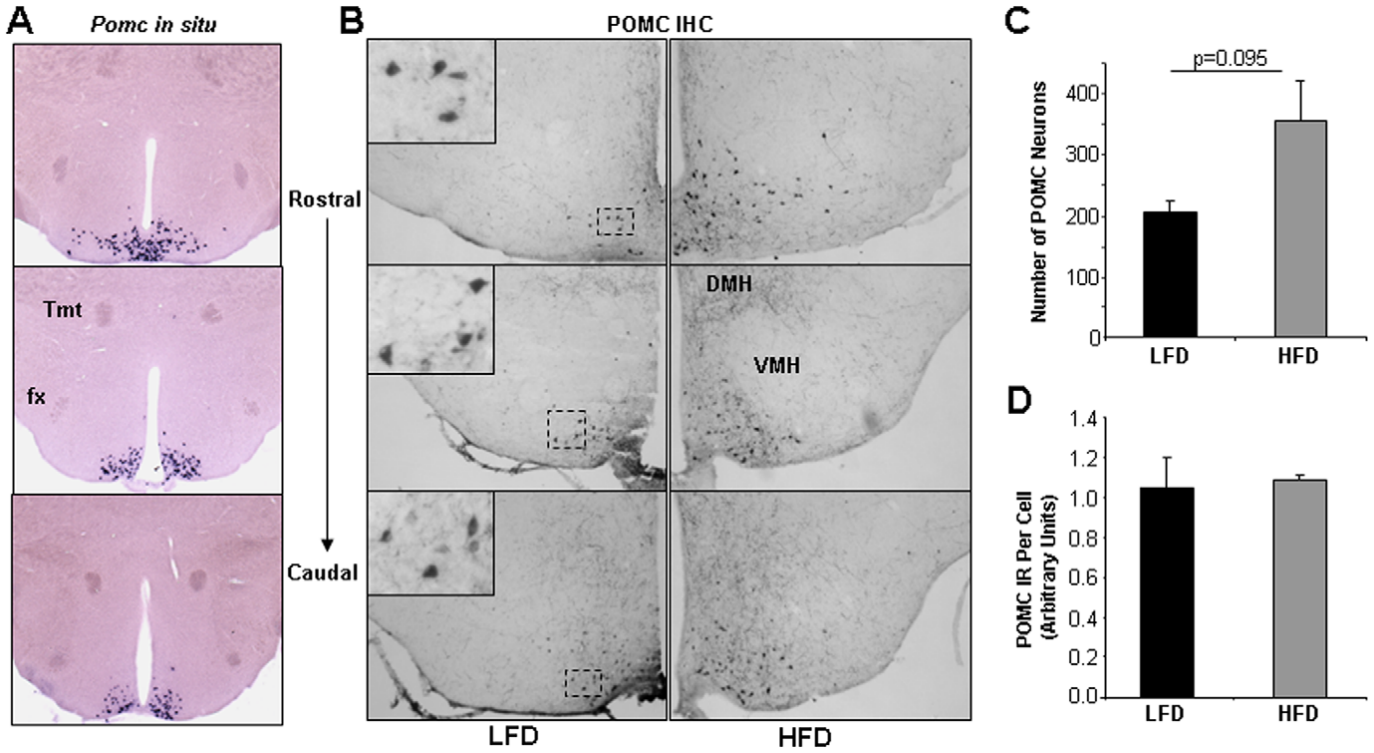
POMC polypeptide expression in LFD and HFD C57Bl/6J mice. A. Shown are matched representative images of POMC neurons as detected in situ hybridization. Matched coronal sections from the rostral, medial and caudal mediobasal hypothalamus of a LFD mouse are presented. B. Shown are matched representative images POMC neurons as detected by POMC immunohistochemistry in the mediobasal hypothalamus of LFD and HFD mice. Matched coronal sections from the rostral, medial and caudal mediobasal hypothalamus are presented. C57Bl/6J mice were given diets for 10 weeks and injected with leptin (0.6 mg/kg) 30 minutes before sacrifice. C. Number of POMC-immunoreactive neurons per LFD and HFD mouse. N = 2 mice per group. D. Shown is quantification of POMC immunoreactivity per POMC neuron. N = 2 mice per group. Tmt = Mammillothalamic tract; fx = fornix; DMH = Dorsomedial hypothalamic nucleus; VMH = Ventromedial hypothalamic nucleus. Data are means +/− SD.

**Figure 5:**
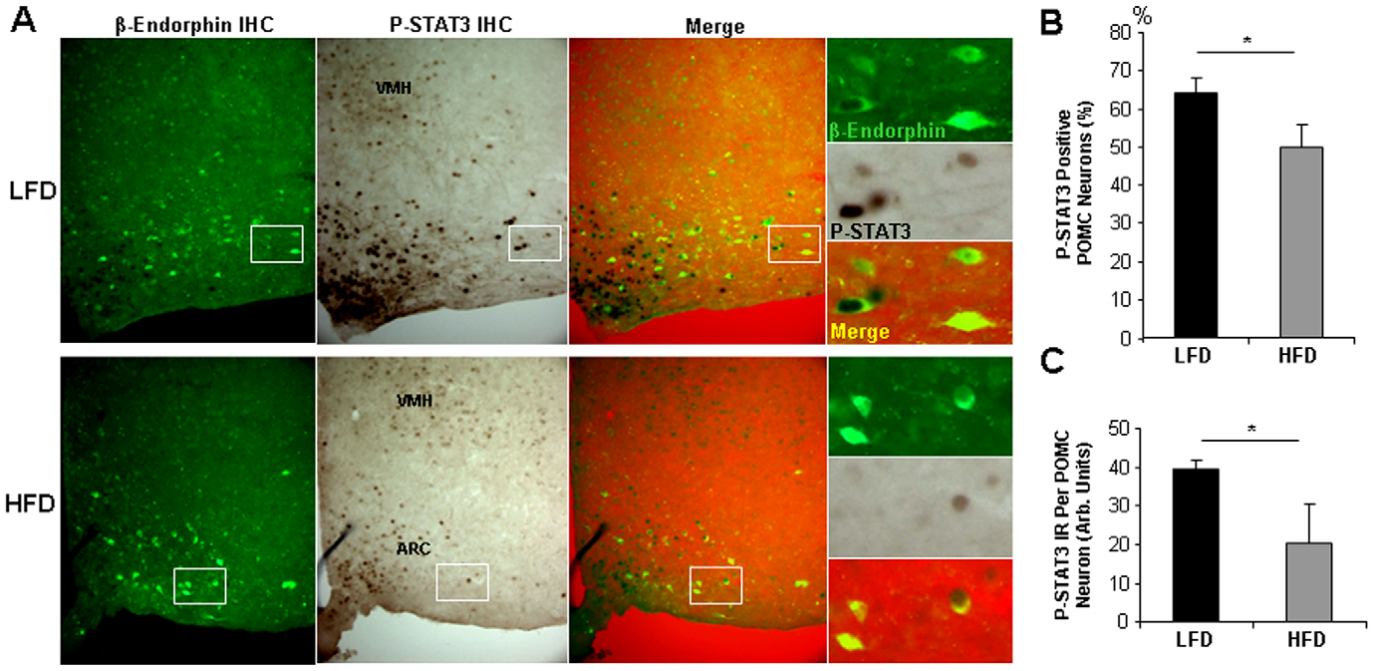
Leptin-dependent STAT3 phosphorylation in POMC neurons of LFD and HFD C57Bl/6J mice. A. Shown are representative images of double immunohistochemistry for P-STAT3 (DAB) and β-Endorphin (green fluorescence) in the mediobasal hypothalamus of leptin-treated LFD and HFD mice. C57Bl/6J mice were given diets for 16 weeks and injected with leptin 30 minutes before sacrifice. B. Percentage of P-STAT3 immunoreactive POMC (β-Endorphin) neurons per hypothalamus. One hemisphere of 9–11 matched hypothalamic brain sections was analyzed in each animal. C. Shown is the total P-STAT3 immunoreactivity (P-STAT3 IR) in all POMC neurons. For B. and C. P-STAT3 was counted and quantified in 258±24 POMC neurons from LFD mice (N = 3) and in 289±25 POMC neurons from HFD mice (N = 4). VMH = Ventromedial hypothalamic nucleus; DMH = Dorsomedial hypothalamic nucleus. * = p<0.05. Data are means +/− SEM.

A change in the number of key leptin-responsive neurons would likely affect whole-body leptin action and therefore represents on possible mechanism causing leptin-resistant DIO. We therefore assessed the number of POMC neurons in lean and obese mice. In 4-week LFD C57Bl/6J mice (N = 4), we found by *in situ* hybridization (ISH) a total of 3310±360 POMC neurons in the entire hypothalamus (Fig. 4A). In HFD mice (N = 4), the number of POMC neurons as determined by ISH was slightly increased by 125% to 4100±260, although this difference was not statistically significant (p = 0.063). The number of POMC neurons as determined by immunohistochemistry (Fig. 4B) also indicated a modest increase (∼175%; p = 0.095) in 10 wk HFD mice (Fig. 4C). POMC polypeptide levels per POMC neuron (soma) were not different between the two groups (Fig. 4D). To specifically assess cellular leptin sensitivity within POMC neurons of DIO mice, we counted POMC neurons that were positive for P-STAT3 (nuclear) by double P-STAT3/β-Endorphin immunohistochemistry (Fig. 5A), according to methods described earlier [16]. As shown in Fig. 5B, the percentage of leptin-responsive (P-STAT3-IR) POMC neurons (β-Endorphin-IR) was reduced from 64% in lean LFD mice to 50% in obese HFD mice (p = 0.024). More strikingly, quantification of the average level of STAT3 phosphorylation (nuclear) within individual POMC neurons showed a reduction by 50% in DIO mice (p = 0.036) (Fig. 5C). Combined, these data suggest that DIO mice are characterized by increased ARC expression of *Socs3* and *Leprb* mRNA (Fig. 2), and by a marked cellular resistance of arcuate neurons (Fig. 3), including specifically POMC neurons (Fig. 5), to activate intracellular LepRb signaling in response to exogenous leptin.

### Diet-induced obesity in C57Bl/6J mice over-expressing leptin receptors in POMC neurons

**Figure 6:**
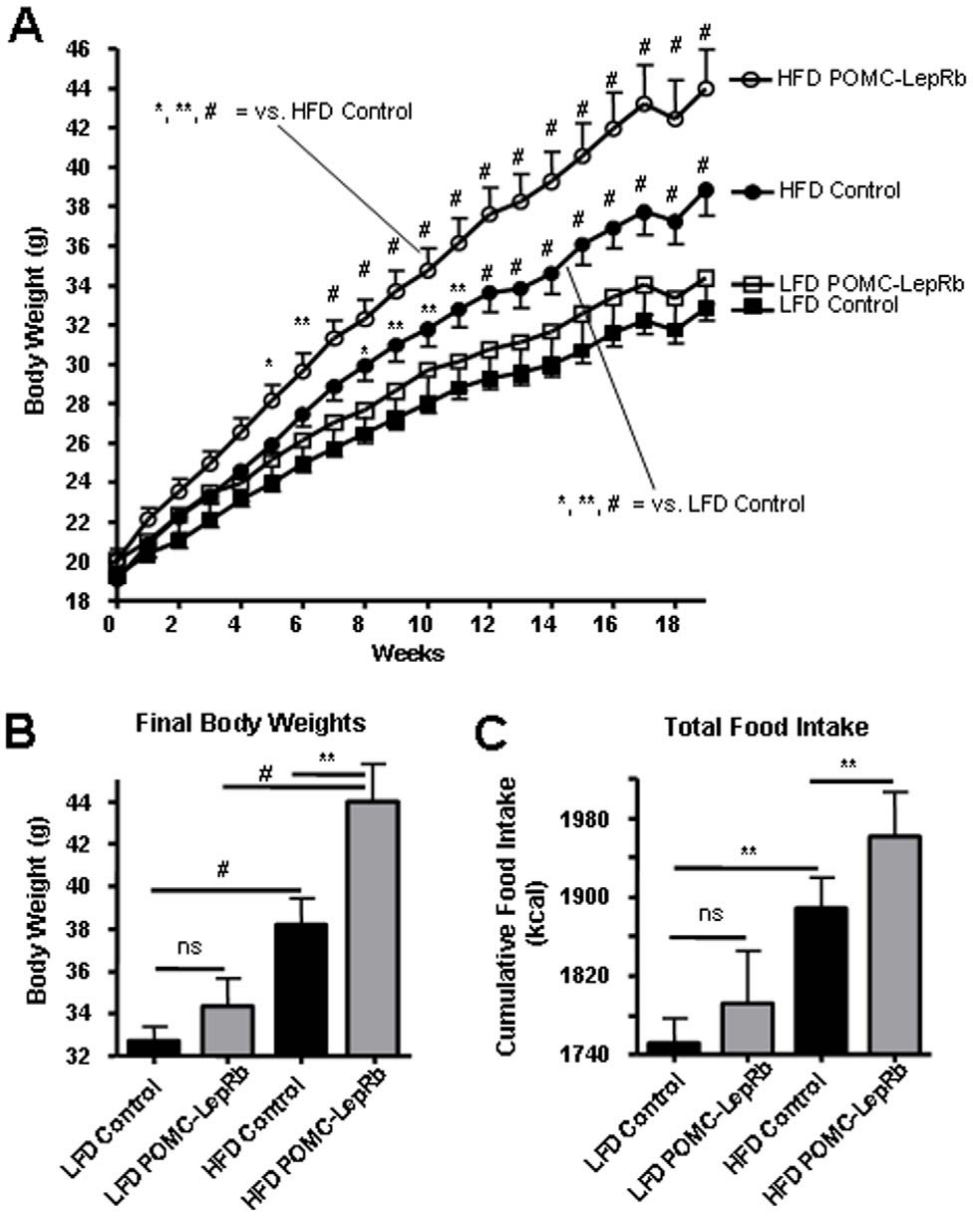
Body weight and caloric intake of POMC-LepRb mice on HFD and LFD. A. Body-weight curves of C57Bl/6J control mice and POMC-LepRb (C57Bl/6J) mice given HFD or LFD diets. B. Final average body weights after 19 weeks on diets. C. Cumulative food intake during the 19 weeks on diets. * = p<0.05; ** = p<0.01; # = p<0.001; ns = Not significant. All mice are littermates. N = 8–9 POMC-LepRb and N = 20–21 control animals per group. Data are means +/− SEM.

**Figure 7:**
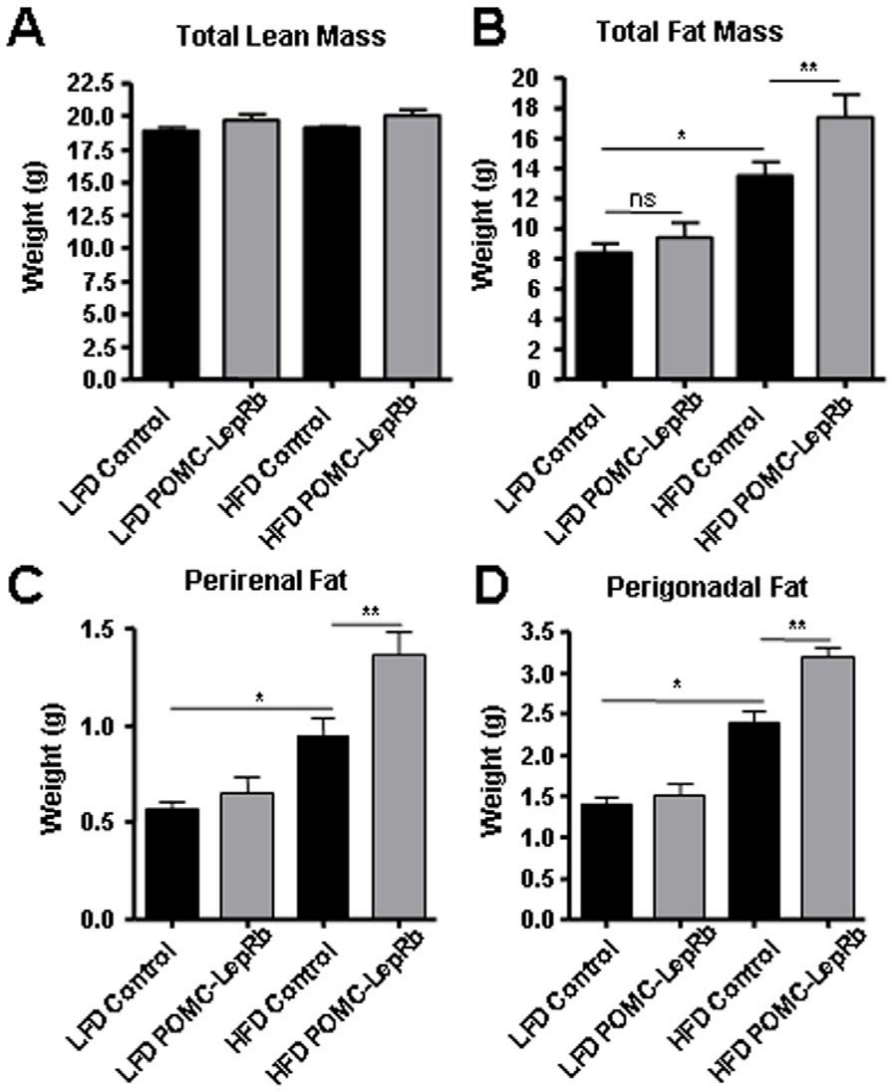
Body composition of POMC-LepRb mice on HFD and LFD. A. Total lean mass. B. Total adipose mass. C. Perigonadal adipose mass. D. Perirenal fat mass. All animals are littermates. * = p<0.05; ** = p<0.01. ns = Not significant. N = 6–7 POMC-LepRb and N = 15–16 control animals per group. Data are means +/− SEM.

**Figure 8:**
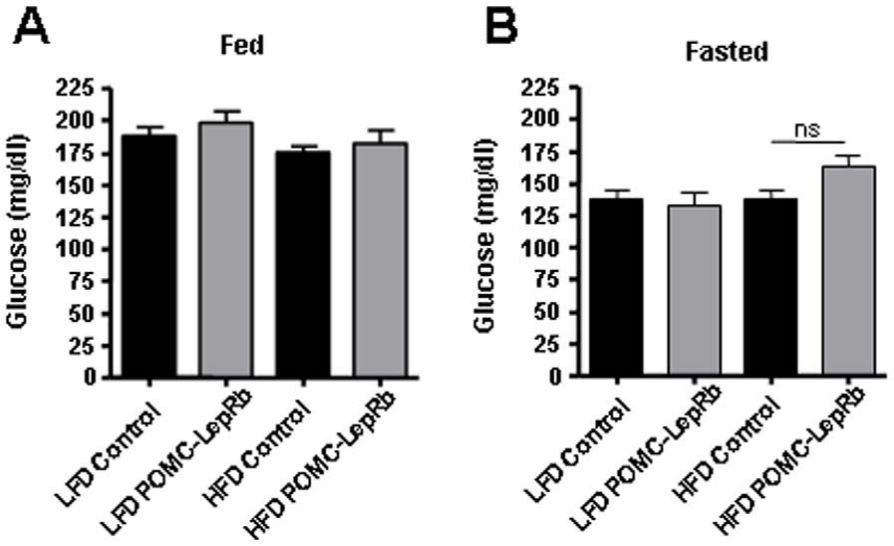
Serum glucose in POMC-LepRb mice on HFD and LFD. A. Ad libitum fed glucose levels. B. Overnight fasted glucose levels. Fed glucose was measured at 12 weeks and fasted levels at 17 weeks on diets. ns = Not significant. N = 8–9 POMC-LepRb and N = 18–19 control mice per group. Data are means +/− SEM.

To mechanistically pursue the above findings and directly investigate the possibility that *increased* LepRb expression in arcuate POMC neurons may play a role in the development of hypothalamic leptin resistance in DIO mice, we tested susceptibility to develop DIO in transgenic mice that over-express HA-tagged LepRb specifically in POMC neurons (POMC-LepRb mice). We have previously created POMC-LepRb mice and demonstrated that HA-LepRb is functional and is selectively expressed in POMC neurons [16]. We here first backcrossed the POMC-LepRb mice from a mixed genetic background to the C57Bl/6J background for better comparison to the above data from C57Bl/6J DIO mice (Fig. 1, 2, 3, 4, 5), since it is well known that genetic background greatly affects sensitivity to high-fat feeding. C57Bl/6J POMC-LepRb mice and littermate controls were then given *ad lib* access to the HFD or LFD for 19 weeks. As shown in Fig. 6A, and as expected, C57Bl/6J control mice on the HFD gained weight faster as compared to C57Bl/6J mice maintained on the LFD. Furthermore, the body-weight curve of POMC-LepRb mice given the LFD was not different from that of littermate controls on the same LFD. This is consistent with our previous data showing that the BW of POMC-LepRb mice (both on a pure FVB genetic background and on a mixed FVB/C57Bl6J/KsJ background) are not different from the BW of littermate controls on a chow diet [16]. Interestingly however, when C57Bl/6J POMC-LepRb mice were given the HFD, BW increased markedly faster relative to C57Bl/6J control littermates on the same diet (Fig. 6A). The increased BW of HFD POMC-LepRb animals correlated with increased cumulative caloric intake (Fig. 6B and 6C), and with increased total fat mass and weights of select adipose depots (Figs. 7B–D). Lean mass (Fig. 7A) and blood glucose concentrations (Fig. 8) did not differ between groups.

**Figure 9:**
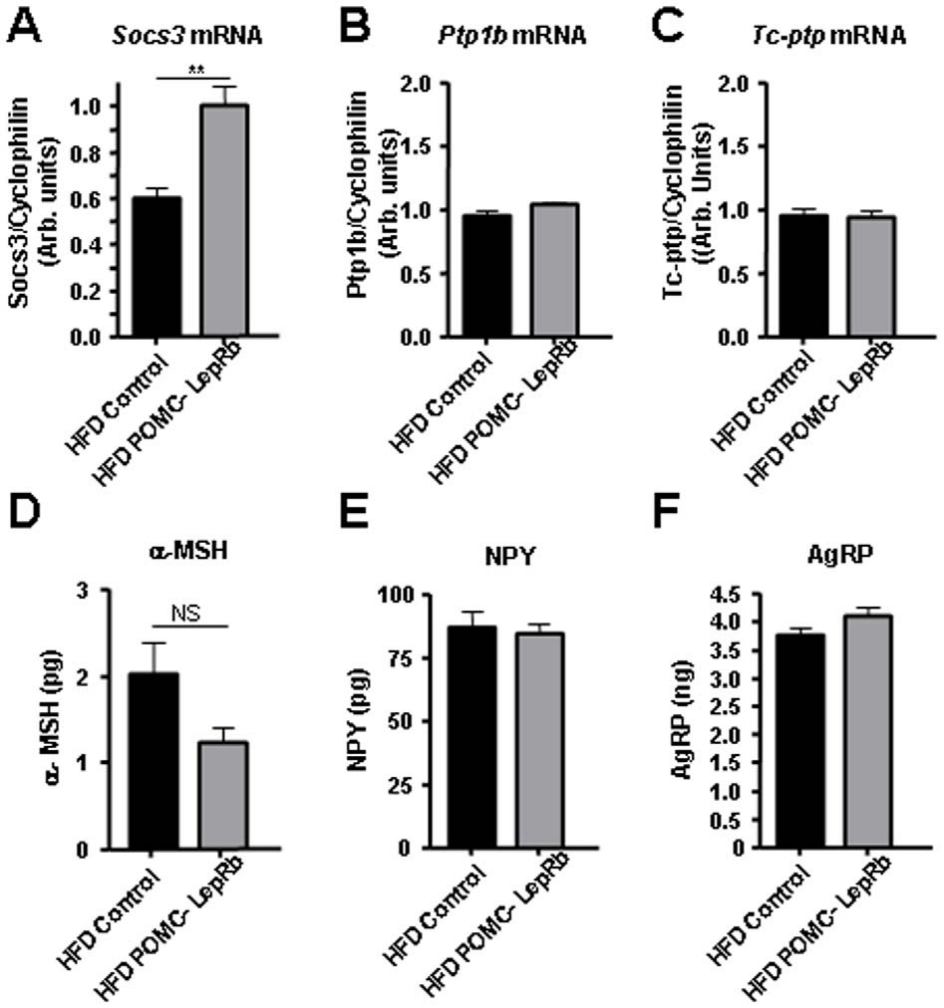
Arcuate mRNA and hypothalamic neuropeptide levels in HFD POMC-LepRb mice. A–C. Arcuate SOCS3 mRNA, PTP1B mRNA and TC-PTP mRNA in POMC-LepRb and control mice after 19 weeks of HFD. Each mRNA was normalized to cyclophilin mRNA in the same samples. N = 6 POMC-LepRb and N = 11 control mice. D-F. Whole hypothalamic α-MSH, NPY and AgRP neuropeptide levels in POMC-LepRb and control mice after 19 weeks of HF diet. α-MSH: N = 6 POMC-LepRb and N = 13 control mice. NPY and AgRP: N = 3 POMC-LepRb and N = 10 control mice. ** = p<0.01. Data are means +/− SEM.

Analyses of ARC mRNA in HFD POMC-LepRb mice revealed that *Socs3* expression was increased (∼60%, p<0.01) relative to HFD controls, while mRNA levels of *Ptp1b/Ptpn1* and *Tc-ptp/Ptpn2* were not altered (Figs. 9A–C). Hypothalamic NPY or AgRP neuropeptide levels were also not different between the two groups. α-MSH neuropeptide levels in the hypothalamus of HFD POMC-LepRb mice were not statistically different (p = 0.16) from that of HFD controls (Figs. 9D–F).

**Figure 10:**
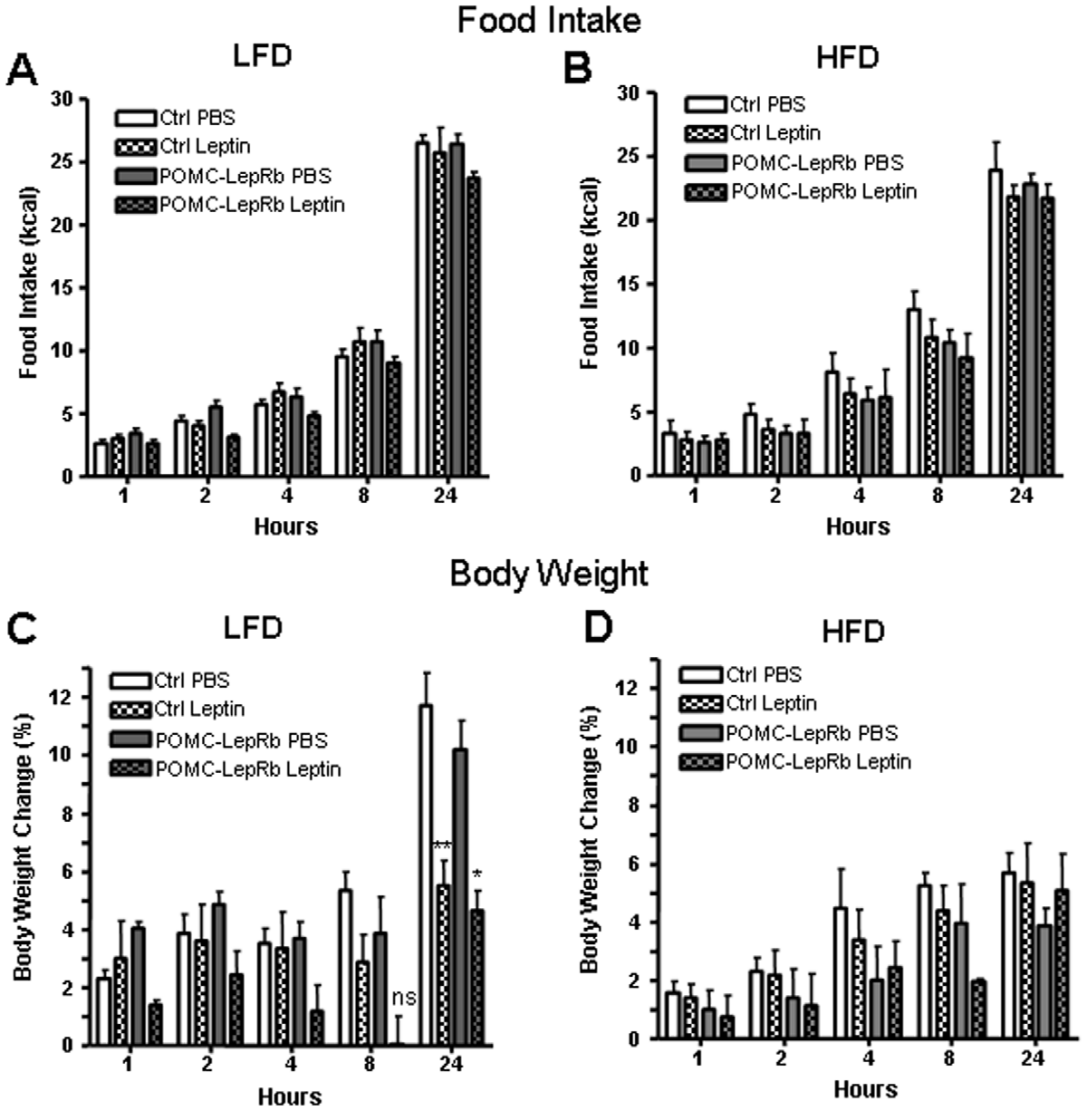
Leptin-sensitivity in POMC-LepRb mice on HFD and LFD. A. Acute food intake after leptin (5 mg/kg, i.p.) or vehicle (PBS) in POMC-LepRb and control mice on LFD. B. Body weight change (relative to preinjection weight) after leptin administration in POMC-LepRb and control mice on LFD. C. Food intake after leptin in POMC-LepRb and control mice after 5 weeks on HFD. B. Body weight change after leptin in POMC-LepRb and control mice after 5 weeks on HFD. N = 4 POMC-LepRb and N = 5 control mice per group. * = p<0.05; ** = p<0.01; ns = not significant. Data are means +/− SEM.

**Figure 11:**
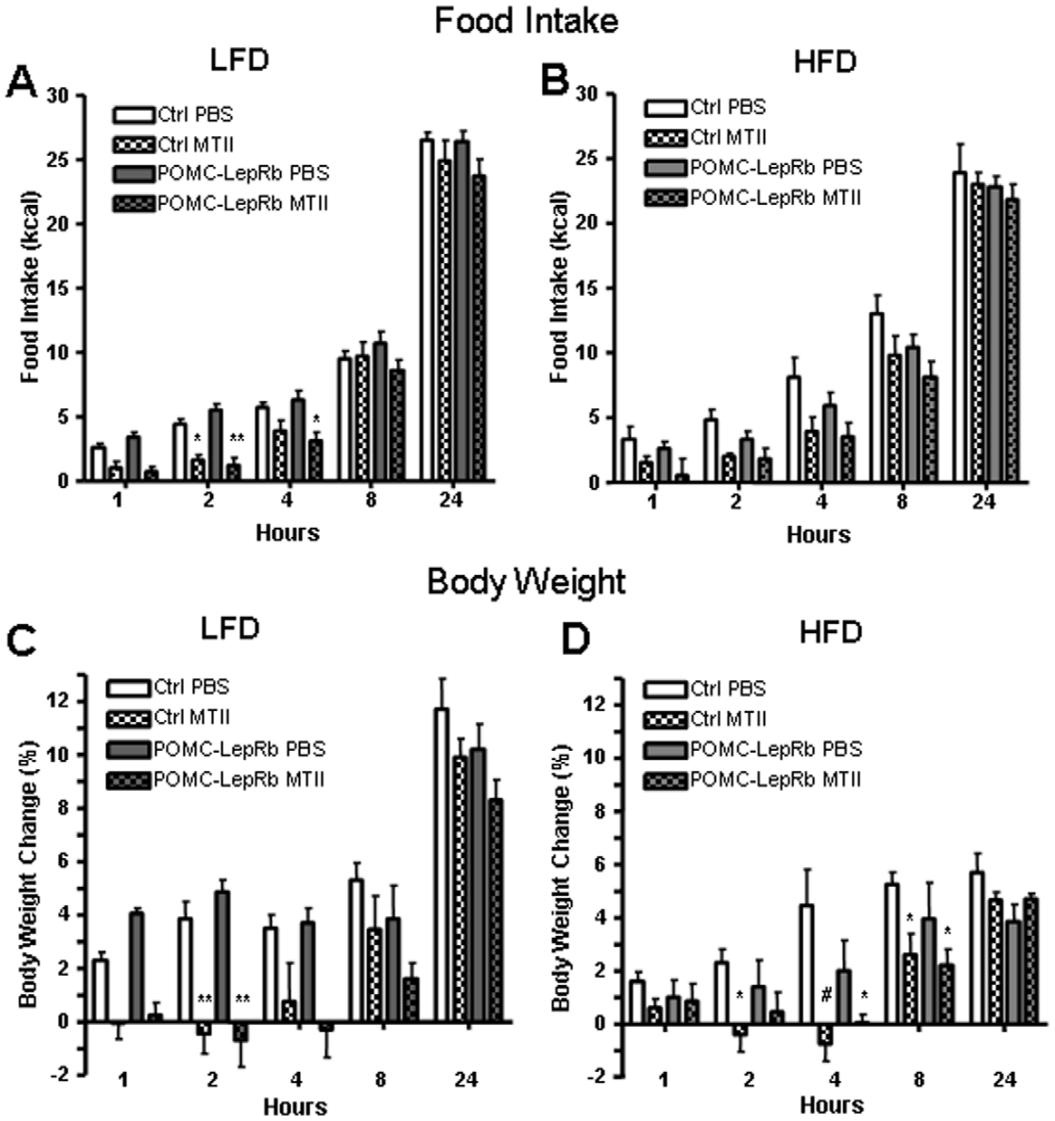
Melanocortin (MTII)-sensitivity in POMC-LepRb mice on HFD and LFD. A. Acute food intake after MTII (5 mg/kg, i.p.) or vehicle in POMC-LepRb and control mice on LFD. B. Body weight change (relative to preinjection weight) after MTII administration in POMC-LepRb and control mice on LFD. C. Food intake after MTII in POMC-LepRb and control mice after 5 weeks on HFD. B. Body weight change after MTII in POMC-LepRb and control mice after 5 weeks on HFD. N = 4 POMC-LepRb and N = 5 control mice per group. * = p<0.05; ** = p<0.01; # = p<0.001. Data are means +/− SEM.

To assess whole-body leptin-sensitivity, POMC-LepRb and control mice were injected with leptin or vehicle (PBS) before and after 5 weeks of HFD. This dose of leptin (5 mg/kg) did not significantly affect 1–24 hour food intake in LFD or HFD controls or in POMC-LepRb mice on either diet (Figs. 10A and 10B), although there was a trend towards lower intake at all time-points in POMC-LepRb mice on the LFD, but not on the HFD. Leptin significantly reduced 24-hour BW-gain in both LFD control and LFD POMC-LepRb mice (Fig. 10C). Leptin did not affect food intake or BW-gain in any of the two groups of mice on the HFD (Fig. 10D), indicative of leptin resistance. To assess responsiveness to anorexigenic signals immediately down-stream of POMC neurons, effects of MTII, a stable α-MSH analogue and agonist of the central melanocortin receptors, on BW and energy intake were measured. As shown in Fig. 11A, MTII reduced intake (2–4 hours) similarly in both control and POMC-LepRb mice given a LFD. MTII's food-inhibitory effect was not significant in either group on the HFD. However, MTII inhibited BW-gain in both groups given either the LDF or HFD (Figs. 11C and 11D), altogether suggesting lack of leptin resistance in neuro-circuitries (the melanocortin pathway) located immediately downstream of POMC neurons of DIO mice.

## Discussion

There are two primary findings in this report. First, we show that POMC neurons of DIO mice are resistant to activation of STAT3 phosphorylation by leptin. Secondly, we find that over-expression of LepRb selectively in hypothalamic POMC neurons is sufficient to accelerate development of diet-induced obesity. We propose a model where over-reactivity of the leptin-LepRb signaling system in POMC neurons may play a role in development of neuronal leptin-resistance and obesity of high-fat diet fed mice.

Several groups, including our own, have previously reported impaired activation by leptin of STAT3 DNA-binding activity and of STAT3 phosphorylation within the hypothalamus, particularly the arcuate nucleus, of DIO mice [30], [31], [47], [50], [51]. However the neuro-chemical nature of those P-STAT3 resistant cells has not been reported. Our identification of arcuate POMC neurons of DIO mice as being resistant to STAT3 phosphorylation by leptin at least opens the possibility that those specific cells might play a causal role in the development of hyperphagia and diet-induced obesity. Since the extent of impaired neuronal P-STAT3 activation is observed throughout the arcuate of DIO mice, it is likely that the many if not all of the remaining LepRb-expressing neurons in this nucleus are also leptin resistant, including the AgRP/NPY neurons. This is consistent with the earlier finding that both POMC and AgRP/NPY neurons from DIO mice show impaired neuropeptide release in response to leptin [51].

We further observed that arcuate *Leprb* mRNA is increased in diet-induced obese mice after 11 weeks of HFD. Some studies [29], [44], [45], but not all [31], [51], [52], [53], have similarly reported an increase in *Lepr* mRNA in the ARC of DIO mice. The reason for the divergent results is unclear but is likely due to various lengths of the feeding studies, use of different rodent strains/species or different compositions of diets, and/or whether whole hypothalamic tissue or only arcuate tissue was analyzed. We have not been able to detect LepRb by Western blotting of hypothalamic tissues in order to complement the mRNA data. This is likely due to insufficient expression of the receptor, since we have previously shown that we are able to detect LepRb in protein lysates from transfected cells [17]. The mechanism whereby HFD might increase hypothalamic *Leprb* gene-expression is unknown. Possibilities include that leptin itself (hyperleptinemia) via LepRb signaling directly or alternatively, that a component in the HFD indirectly stimulates hypothalamic *Leprb* mRNA. The increase in arcuate *Leprb* mRNA in DIO mice could indicate the presence of counter-regulatory mechanism. Alternatively, it is possible that up-regulation of leptin receptor expression is a primary event that may cause cellular leptin resistance through chronic over-stimulation the leptin-LepRb signaling system. In support of this possibility, we show here that forced over-expression of LepRb in POMC neurons is sufficient to accelerate weight gain in HFD mice. In addition, hyperleptinemia itself is reported to be required for development of hypothalamic leptin-resistant STAT3 signaling in DIO mice [47].

The result that over-expression of LepRb in POMC neurons increases body weight in HFD mice is initially surprising, considering the well known anti-obesity effect of the leptin-LepRb system and our recent report showing that expression of LepRb in POMC neurons of *Lepr^db/db^* mice reduces body weight [16]. This apparent paradox may be explained by the fact that total LepRb expression within individual POMC neurons is likely higher in our POMC-LepRb mice of current study because of the presence of both transgenically-derived LepRb and of endogenous LepRb. In addition, the HFD may further increase endogenous *Leprb* mRNA in POMC-LepRb mice as discussed above. In contrast, POMC-LepRb-*Lepr^db/db^* mice only express transgenically-derived LepRb proteins (which we have previously shown to be expressed at a level that is similar to that of endogenous LepRb in POMC neurons of normal mice [16]). In addition, in the earlier study we did not challenge POMC-LepRb-*Lepr^db/db^* mice with a HFD. If POMC-LepRb-*Lepr^db/db^* mice do not accelerate weight gain when given a HFD, we would conclude that either the total LepRb expression within POMC neurons level in too low, or alternatively, that leptin signaling in other neurons in addition to POMC neurons is required for development of DIO. These important issues will be investigated in future studies.

On the high-fat diet, we found that the POMC-LepRb animals were similarly resistant to exogenous leptin as compared to HFD control mice. In contrast, POMC-LepRb mice on LFD and HDF were similarly sensitive to MTII-induced body weight loss as compared to control mice. These data combined at least suggest that the site of leptin resistance in the HFD POMC-LepRb mice is at the level of the POMC neurons themselves, and not in downstream pathways.

The exact mechanism whereby a HFD causes chronic up-regulation of arcuate *Socs3* expression in DIO mice is unclear. In lean rodents, leptin acutely stimulates STAT3 phosphorylation and *Socs3* mRNA in POMC neurons [14], [35], [54]. Indeed, P-STAT3 stimulates *socs3* gene expression via direct binding to the *socs3* promoter [20], [21]. SOCS3 proteins then act in a negative feedback loop to attenuate leptin signaling [2], [19]. In cell lines, acute leptin signaling can induce long-lasting SOCS3 protein expression and leptin resistance (i.e. impaired STAT3 activation) [46]. In the ARC of normal DIO mice, *Socs3* mRNA is chronically elevated [31], [51] and neurons are resistant to STAT3 activation by leptin [31], [50], [51], [55], [56]. One possible mechanism explaining the chronic increase in *Socs3* and impaired leptin signaling is that the HFD, either directly via components in the diet itself or indirectly by increasing local or systemic levels of inflammatory cytokines, stimulates arcuate *Socs3* gene-expression [34], [57], [58], [59], [60]. Alternatively, hyperleptinemia itself may increase *Socs3* expression to a level that eventually inhibits LepRb-STAT3 signaling, similar to what we have shown earlier in leptin-treated cell lines [46]. Our current data are consistent with a model where the HFD increases arcuate LepRb expression resulting in over-stimulation of LepRb signaling pathways, ultimately leading to long-term elevation of SOCS3 proteins levels and chronic down-regulation of LepRb signaling. Indeed, mice that chronically over-express leptin accumulate fat mass with age [48] and exhibit increased susceptibility to diet-induced obesity [49].

Consistent with this model of over-stimulation of LepRb signaling in POMC neurons leading to obesity, it has been reported that forced over-expression of a constitutively active (CA) form of STAT3 in POMC neurons is sufficient to cause obesity, even in mice given a chow diet [55]. These CA STAT3-POMC animals have chronically elevated hypothalamic *Socs3* mRNA, presumably mediated by constitutively active STAT3-induced *Socs3* gene-expression in POMC neurons. It is therefore possible that SOCS3 protein levels have reached an “obese threshold” in POMC neurons in those mice, causing reduced LepRb-STAT3 signaling. The reason that our LepRb-POMC mice do not develop obesity on a LFD (Fig. 6) or chow diet [16], in contrast to the CA STAT3-POMC mice, may be explained by the lack of elevated leptin levels; i.e. despite the forced increase of LepRb, the circulating leptin concentration is not sufficient to activate STAT3 and to increase *Socs3* expression to the threshold-level that causes long-term cellular leptin-resistance. We propose that any mechanism that increases SOCS3 protein expression beyond a certain level in POMC neurons will cause long-term leptin resistance in those cells and eventually to obesity. Consistent with this “SOCS3-threshold” hypothesis, it was recently demonstrated that genetically-driven over-expression of SOCS3 in POMC neurons is sufficient to cause obesity even in chow-fed mice [36]. In addition, genetic deletion of *Socs3* from POMC neurons attenuates the development of DIO [35]. Importantly, since these POMC-SOCS3 KO mice given a HFD still show an increase in body weight and hyperleptinemia compared to LFD mice (albeit both are attenuated compared to HFD control mice), it is clear that additional mechanisms (e.g. elevated LepRb and/or SOCS3 (and/or PTP1B and/or PTPN2) expression in other neurons (e.g. AgRP)) also play important roles in the development of leptin resistance and diet-induced obesity.

In conclusion, we show that POMC neurons of DIO mice are resistant to STAT3 activation by leptin and that over-expression of LepRb only in POMC neurons is sufficient to potentiate the development of diet-induced obesity. We propose a model where chronic over-stimulation of the leptin-LepRb signaling pathway in arcuate neurons may play a role in development of arcuate leptin resistance and diet-induced obesity, possibly via elevation of SOCS3 expression.

## Materials and Methods

### Ethics Statement

Care of mice and animal procedures used in this study were approved (protocol #011-2010) and performed according to standards set by the Institutional Animal Care and Use Committee (IACUC) at Beth Israel Deaconess Medical Center.

### Animals and animal care

Male C57Bl/6J mice at 3–4 weeks of age were ordered from Charles River Laboratories (Boston, MA). Transgenic POMC-LepRb mice on the C57Bl/6J background were created as described below. Care of mice and animal procedures were approved and performed according to standards set by the Institutional Animal Care and Use Committee (IACUC) at Beth Israel Deaconess Medical Center. Unless otherwise noted, mice were housed at 22°C–24°C using a 14 hr light/10 hr dark cycle with light on a 7 a.m. and chow food (Teklad F6 Rodent Diet 8664, 4.05 kcal/g, 3.3 kcal/g metabolizable energy, 12.5% kcal from fat, Harlan Teklad, Madison, WI) and water provided *ad libitum*.

### Diet-induced obesity

At 5-weeks of age, mice were placed on a low-fat diet (LFD) (Research Diets 12329: 16% protein; 73% carbohydrate; 11% kcal from fat) or a high-fat diet (HFD) (Research Diets 12331: 16% protein; 26% carbohydrate; 58% kcal from fat, Research Diets, New Brunswick, NJ). Body weight (BW) was measured once a week. For food-intake studies, mice were housed individually. Fresh pellets of food were provided every 3 days to avoid temperature-dependent spoilage of the HFD, and cages were changed every time food weight was measured. Any residual bits of food in the bedding were included in measurements. Cumulative food intake data was obtained by adding all intake measurements during the study.

### Generation of C57Bl/6J POMC-LepRb mice

*HA-LepRb STOP* transgenic mice [16] were mated with wild-type C57Bl/6J mice (Charles River Laboratories) for seven generations. *Pomc-Cre* mice, also described earlier [14], were kindly supplied by Dr. Bradford Lowell (BIDMC, Boston, MA) and also mated with C57Bl/6J mice for at least seven generations. The C57Bl/6J *HA-LepRb STOP* (heterozygous) mice were then mated with C57Bl/6J *Pomc-Cre* (heterozygous) mice to generate C57Bl/6J *Pomc-Cre/HA-LepRb* mice (*POMC-LepRb*) and control littermates.

### Blood composition

Tailvein blood was collected at 12:00 PM±2 hr from mice maintained on LFDs or HFDs. Mice were either *ad libitum* fed or fasted for 24 hours. At 12 and 17 weeks of age, blood was assayed for fed and fasted glucose levels, respectively (Fisher Scientific, Morrison Plains, NJ).

### Isolation of hypothalamic tissues by microdissection

Animals were euthanized by cervical dislocation and brains were rapidly removed. Using a cooled mouse brain matrix with 1-mm section dividers (ASI Instruments Inc., Warren, MI), one sagittal cut was made to bisect the brain, followed by two cuts left and right of the bisectional cut to produce two 1-mm-thick sagittal sections left and right of the third ventricle. Landmarks including the fornix, optic tracts and mammillary nuclei were used to dissect reproducible pieces of the ARC-enriched regions, as described earlier [31]. Two tissue pieces (one from each hemisphere) from each animal were combined and snap-frozen in liquid nitrogen and stored at −80°C until further use.

### RNA extraction, cDNA synthesis and real-time PCR

Total RNA was isolated from ARC tissue blocks using RNA STAT60 (Tel-Test, Friedenswood, TX). Five hundred nanograms of total RNA was used for reverse transcription (RT-PCR kit; Clonetech, Palo Alto, CA). Quantification was carried out by real-time PCR using the Stratagene Mx3000P system. The real-time PCR was performed according to the manufacturer's instructions with minor alterations. The primers (F and R) (Invitrogen, Carlsbad, CA) and probes (P)(Biosearch Technologies, Novato, CA) were designed with the assistance of PrimerExpress software as follows: SOCS3F (5′-GCGGGCACCTTTCTTATCC-3′), SOCS3R (5′-TCCCCGACTGGGTCTTGAC-3′) and SOCS3P [5′- TCGGACCAGCGCCACTTCTTCAC-BHQ-1–3′); CyclophilinF (5′- GGTGGAGAGCACCAAGACAGA-3′), CyclophilinR (5′-GCCGGAGTCGACAATGATG-3′) and CyclophilinP (5′-ATCCTTCAGTGGCTTGTCCCGGCT-3′). Primers/Probe sets for *Pomc* were: POMCF (5′-ACCTCACCACGGAGAGCA-3′), POMCR (5′-GCGAGAGGTCGAGTTTGC-3′) and POMCP (5′-TGCTGGCTTGCATCCGGG-3′); for *Npy*: NPYF (5′-CTCCGCTCTGCGACACTAC-3′), NPYR (5′-AATCAGTGTCTCAGGGCT-3′) and NPYP (5′-CAATCTCATCACCAGACAG′-3); and for *Agrp*: AgRPF (5′-GCGGAGGTGCTAGATCCA-3′), AgRPR (5′-AGGACTCGTGCAGCCTTA-3′) and AgRPP (5′-CGAGTCTCGTTCTCCGCG-3′). Primers/Probe sets for *Leprb Jak2*, *Socs1*, *Ptp1b* and *Tc-ptp* were purchased from Applied Biosystems (Foster City, CA). PCRs were run in a volume of 25.0 µl using 1.0 µl cDNA. A standard curve was generated from duplicate measurements of serial dilutions of ARC cDNA.

### Quantification of neuropeptides

Neuropeptides were extracted from whole hypothalamic tissues and measured by enzyme immunoassay (EIA). The extraction and purification of the peptides were performed as previously described [16]. The samples were then assayed by EIA for α-MSH, NPY, and AgRP (all in-house assays) [16]. The sensitivities of the assays were 2, 8 and 9 pg per sample, respectively.

### Leptin and MTII sensitivity

Mice were fasted overnight and given vehicle (PBS) or leptin (5 mg/kg)(A.F. Parlow National Hormone and Peptide Program, Torrance, CA) or Melanotan II (MTII) (5 mg/kg)(Tocris, Ellisville, MO). Baseline body weight was measured and a known amount of food was added to each cage. Body weight and food intake were then measured 1, 2, 4, 8, and 24 hours post-injection.

### Immunohistochemistry (IHC)

Five series of 25 µm coronal brain sections were generated from each animal as described earlier [16]. Supplies were from Sigma-Aldrich (St. Louis, MO) and the ABC Vectastain Elite kit from Vector Laboratories (Burlingame, CA). The phospho-specific-(Y705)-STAT3 rabbit antibody was from New England Biolabs (Beverly, MA), the rabbit anti-POMC antibody was from Phoenix Pharmaceuticals (Burlingame, CA) and the rabbit anti-β-endorphin antibody was a kind gift from Dr. Oline Ronnekleiv (Oregon Health and Science University, Portland, OR). The biotinylated donkey-anti-rabbit antibody was from Jackson Immunology Research Laboratories (West Grove, PA). Fluorescent donkey anti-rabbit immunoglobulin conjugates were from Molecular Probes (Eugene, OR), and donkey serum was from Invitrogen Life Technologies, Inc (Carlsbad, CA). POMC- and P-STAT3-immunoreactivity was detected by 3,3′-diaminobenzidine (DAB) and β-endorphin by fluorescence.

### Quantification of leptin-induced nuclear STAT3 phosphorylation in arcuate neurons

Mice that were maintained on a LFD or HFD for 10 weeks were injected i.p. with leptin (0.6 mg/kg (half-max dose); 30 min). Brain sections (25 µm) were processed for P-STAT3 using the ABC detection method [31]. The DAB reactions were terminated in parallel for all sections before reaching maximal staining intensity. Bright-field images (20× objective) from 5 matched (rostral-caudal) hypothalamic sections from a 1∶5 series in each animal (N = 3 LFD and N = 3 HFD) were captured with digital camera (AxioCam, Carl Zeiss, Thornwood, NY) mounted on a Zeiss microscope (Axioscope2). P-STAT3 immunoreactive ARC neurons were counted and nuclear-P-STAT3 staining in each cell was quantified (grey-scale density) using ImageJ software (NIH) [16] Cells within the median eminence and in non-arcuate nuclei were excluded from these analyses. One brain hemisphere was analyzed in each section.

### Quantification of POMC polypeptide expression in POMC cell bodies

Brain sections from the 10-week DIO study (above) were processed for POMC IHC. The DAB reactions were stopped in parallel for all sections before reaching maximal staining intensity, which was determined by visual inspection during color development. Brightfield images (20× objective) from 10–11 matched (rostral-caudal) hypothalamic sections from a 1∶5 series in each animal (N = 2 LFD and N = 2 HFD) were captured as described above. POMC-positive neurons throughout the mediobasal hypothalamus were counted and quantified (grey-scale density) using ImageJ software. One brain hemisphere was analyzed in each section.

### Quantification of leptin-induced nuclear STAT3 phosphorylation in POMC neurons

Mice from a separate 16-week long DIO study were injected i.p. with leptin (4 mg/kg; 30 min). Brain sections were subjected in parallel to double IHC for P-STAT3 (DAB) and β-Endorphin (fluorescence), as we have described earlier [16]. The DAB reactions were stopped in parallel for all sections before reaching maximal staining intensity as determined by visual inspection during color development. Matching bright-field and fluorescent images (20× objective) from matched hypothalamic sections (N = 9–11) from a 1∶5 series in each animal were captured as described above. POMC neurons were identified based on the cytoplasmic localization of β-Endorphin. Nuclear P-STAT3 staining (grey-scale density) was then quantified with the ImageJ software in each POMC neuron. One brain hemisphere was analyzed in each section.

### *Pomc* in situ hybridization

Free-floating brain sections were generated as described for IHC (above). One 1∶5 series from each animal (4 week-long DIO study) were mounted on Superfrost Plus slides (Fisher, Hampton, NH) and hybridized overnight with a 580 base long digoxigenin (DIG)-labeled mouse *pomc* anti-sense RNA probe (0.6 µg/ml) at 60°C. All sections were washed twice in 0.2X SSC at 60°C, blocked in PBS with 10% bovine serum, and reacted with anti-DIG antibodies fused to alkaline phosphatase (Roche, Nutley, NJ) (1∶5000, 10% serum, 2 hours at room temperature). Sections were washed and incubated with alkaline phosphatase substrate (NBT/BCIP, Roche, Nutley, NJ) producing a color precipitate. The reaction was stopped in parallel for all sections by EDTA before reaching maximal staining intensity as determined by visual inspection during color development. One hemisphere was counted in all hypothalamic sections containing POMC neurons (N = 8–12 per animal). The number of POMC neurons counted in one hemisphere of a complete 1∶5 series was multiplied by 10 (2×5) for determination of the total number of hypothalamic POMC neurons.

### Body composition

Body composition analysis was carried out at 17 weeks of diets using Echo-MRI (Echo Medical Systems, Houston, TX). Wet fat pad weights of individual depots were measured immediately after sacrifice after 19 weeks of diets.

### Data analysis

Data sets were analyzed for statistical significance using two-tailed Student's T-test (Figs. 1A–F, 2, 3B–C, 4C–D, 5B–C, 6A, 9A–F) or using one-way ANOVA (Tukey's posthoc analysis) (Figs. 6B–C, 7A–D, 8A–B) or using two-way repeated measures ANOVA (Bonferroni post-hoc analysis) (Figs. 6A, 10A–D, 11A–D). All parameters are expressed as mean ± SEM. A p-value of <0.05 was accepted as being statistically significant.
